# Process evaluation of two home-based bimanual training programs in children with unilateral cerebral palsy (the COAD-study): protocol for a mixed methods study

**DOI:** 10.1186/s12887-018-1111-1

**Published:** 2018-04-24

**Authors:** Laura Beckers, Jan van der Burg, Yvonne Janssen-Potten, Eugène Rameckers, Pauline Aarts, Rob Smeets

**Affiliations:** 1 Department of Rehabilitation Medicine, School for Public Health and Primary Care (CAPHRI), Maastricht University, Maastricht, the Netherlands; 2 Centre of Expertise in Rehabilitation and Audiology, Adelante, Hoensbroek, the Netherlands; 3 Department of Paediatric Rehabilitation, Sint Maartenskliniek, Nijmegen, the Netherlands; 4 School of Pedagogical and Educational sciences, Radboud University, Nijmegen, the Netherlands; 5 Master of Specialised Physical Therapy, AVANS Plus, Breda, the Netherlands; 6 Libra Rehabilitation and Audiology, Eindhoven/Weert, the Netherlands

**Keywords:** Cerebral palsy, Process evaluation, Mixed methods, Complex intervention, Home program, Bimanual training, Upper extremity, Explicit motor learning, Implicit motor learning, Parental stress

## Abstract

**Background:**

As part of the COAD-study two home-based bimanual training programs for young children with unilateral Cerebral Palsy (uCP) have been developed, both consisting of a preparation phase and a home-based training phase. Parents are coached to use either an explicit or implicit motor learning approach while teaching bimanual activities to their child. A process evaluation of these complex interventions is crucial in order to draw accurate conclusions and provide recommendations for implementation in clinical practice and further research. The aim of the process evaluation is to systematically assess fidelity of the home-based training programs, to examine the mechanisms that contribute to their effects on child-related and parent-related outcomes, and to explore the influence of contextual factors.

**Methods:**

A mixed methods embedded design is used that emerges from a pragmatism paradigm. The qualitative strand involves a generic qualitative approach. The process evaluation components fidelity (quality), dose delivered (completeness), dose received (exposure and satisfaction), recruitment and context will be investigated. Data collection includes registration of attendance of therapists and remedial educationalists to a course regarding the home-based training programs; a questionnaire to evaluate this course by the instructor; a report form concerning the preparation phase to be completed by the therapist; registration and video analyses of the home-based training; interviews with parents and questionnaires to be filled out by the therapist and remedial educationalist regarding the process of training; and focus groups with therapists and remedial educationalists as well as registration of drop-out rates and reasons, to evaluate the overall home-based training programs. Inductive thematic analysis will be used to analyse qualitative data. Qualitative and quantitative findings are merged through meta-inference.

**Discussion:**

So far, effects of home-based training programs in paediatric rehabilitation have been studied without an extensive process evaluation. The findings of this process evaluation will have implications for clinical practice and further research regarding development and application of home-based bimanual training programs, executed by parents and aimed at improving activity performance and participation of children with uCP.

## Background

Cerebral Palsy (CP) is the most common cause of motor disability in children [1]. The restricted motor function of one upper extremity in children with unilateral CP (uCP) mainly leads to perceived difficulty in performing bimanual activities of daily living [2]. These activity limitations often restrict the children’s level of participation with their peers and family, at school and in leisure activities [3]. Most of these children are enrolled in different kinds of interventions during childhood to improve performance of bimanual activities and to promote participation.

There seems to be consensus among clinicians and researchers on the importance of home-based training programs for children with CP [4]. To allow implementation of these programs within the context of family life, collaborative service delivery is required, meaning families collaborate with professionals in the delivery of treatment. An et al. have defined three main principles of importance to collaborative service delivery in paediatric rehabilitation: family identified needs, shared responsibility, and family empowerment [5]. Taking into account the unique needs of the family promotes parents’ perceptions of shared planning. This shared responsibility is crucial for successful and effective implementation of interventions. Since families and professionals have expertise on different areas, various essential perspectives on the child can be taken into consideration. Because of the parents’ engagement in collaborate service delivery, the empowerment of the family is supposed to be supported [5]. As a result, parents may become less dependent on health care professionals in the treatment of their child’s disability. Moreover, home-based training programs enable children to practice bimanual activities within the specific context of their daily lives. As a result, the neural processing demands during training are equal to the demands while performing the tasks in daily life. Therefore, no transfer of training to a new environment is required [6]. Hence, home-based training programs are expected to be highly effective in improving bimanual performance of activities and maintenance of training effects is more likely to occur.

Although there is evidence that home-based bimanual training programs are effective in improving bimanual performance [7, 8], data from several studies suggest that these programs can have adverse consequences too. In a qualitative study, Peplow and Carpenter showed that parents feel a lot of pressure to adhere to the training, eliciting perceived stress. In addition, ensuring that the child performed the prescribed training activities required time and effort from parents and impacted family relationships [9]. Likewise, Lin et al. reported that home-based training led to dysfunctional parent-child interaction and an increase of parental stress [10]. Since parental stress seems to have a negative impact on adherence, this may also limit the effectiveness of home-based programs [11].

Our research group currently performs the COAD-study (co-creation at hand: the road to independence). For this study, two home-based bimanual training programs for young children with uCP have been developed. Both programs aim to increase bimanual activity performance of the child, without increasing parental stress [12]. To pursue this, the programs differ from existing home-based bimanual training programs in two ways. First, a remedial educationalist or health care psychologist (referred to as ‘remedial educationalist’) collaborates with a paediatric occupational or physical therapist (referred to as ‘therapist’) in the coaching of parents in order to specifically focus on the parent-child interaction. The main aim is to establish a functional parent-child interaction, resulting in optimal adherence to the training with as minimal as possible increase of therapy-related parental stress. Second, as it is not clear what type of learning is most effective, parents in one program are coached to use an explicit motor learning approach while teaching motor activities to their child, whereas parents in the other program are coached to use an implicit motor learning approach. Within both programs, the therapist coaches the parents to provide particular instructions and feedback to their child and to organize the training activities in a specific way [13]. The latter includes for instance the type of object to be used (e.g. shape), the position of the child (e.g. sitting at a table) and the setting (e.g. slipperiness of the working surface). In both programs, parents provide task-oriented and result-oriented instructions and feedback to the child. An example of a task-oriented instruction is to tell the child to grab the jar of peanut butter and open it. Result-oriented feedback is for instance to compliment the child on opening the jar himself. Using the explicit approach, parents give additional instructions and feedback to their child regarding specific motor execution of the task. An example of an explicit instruction is asking the child to grab the jar of peanut butter with the affected hand whilst doing ‘the trick of the thumb’ (i.e. abducting the thumb), and to subsequently squeeze the jar with the affected hand while turning the lid with the non-affected hand. By contrast, using the implicit approach, parents do not give any instructions or feedback regarding motor execution of the task. Instead, parents provoke specific motor execution by the organization of the task, for example by positioning the jar of peanut butter on the affected side to elicit the child to grab it with the affected hand, and by using a sufficiently small-sized jar that can easily be squeezed with the affected hand while opening the lid with the non-affected hand. We hypothesize that by using an explicit approach, parents have to prompt their child frequently to attain the specified way of motor execution of the task, which may result in frustration and conflicts between the child and the parent. This may lead to an increase of perceived parental stress and limited adherence to the training. Moreover, the explicit instruction with regard to specific motor execution of the task has to be remembered by the child during task performance. Hence, working memory demands in the explicit approach are expected to be higher than in the implicit approach. As children with CP often have limited working memory abilities [14], this may cause complications during the training. The implicit approach is expected to have less adverse consequences than the explicit approach, as the parents do not need to prompt their child regarding motor execution of the task and working memory demands are lower.

As part of the COAD-study, the home-based training programs are tested for their effects and compared with each other, using a comparative case series design. Effects are measured on child-related outcomes regarding bimanual activity and participation. Additionally, parent-related effects are investigated, concerning parental stress and empowerment.

In addition to the effect evaluation, a process evaluation is needed, in particular because we consider these home-based training programs to be complex interventions [15]. This assumption is based on four reasons. First, each program comprises multiple components that interact with each other. The components are for example instructional videos to train the parents, a task analysis performed by the therapist, performance of the home-based training by the child and parents, and a phone call from the remedial educationalist to coach the parents. Second, the actions required by both the health care professionals and the parents are numerous as well as difficult. Third, the programs aim to produce change on a range of child-related and parent-related outcomes. Fourth, tailoring of the programs to the individual child and parents is permitted to a relatively large extent.

Results regarding effects of our home-based training programs alone are not sufficient to come to accurate conclusions and recommendations for implementation in clinical practice and further research. For example, in case one or both home-based training programs are found to be successful, it is valuable to know why they are effective as well as whether and how they can be optimized. Likewise, if a program is unsuccessful, it is important to know why it is ineffective or has unanticipated effects. Because of the complexity of the programs, we consider that three aspects are important to investigate specifically. First, evaluation of effects must be related to the evaluation of fidelity, indicating whether the programs were performed (i.e. implemented) as intended within the comparative case series. Second, causal mechanisms should be clarified as far as possible, by exploring which components of the programs did and did not contribute to the effects of the programs. Third, it is relevant to identify what contextual factors with regard to children, parents and health care professionals are associated with possible variation in implementation and outcomes between child-parents triads and corresponding health care professionals [15]. In conclusion, a process evaluation of the home-based training programs is a crucial addition to the evaluation of their effects.

Consequently, this study aims to systematically evaluate the processes and factors which influence implementation and effects of the programs. For this purpose, a process evaluation of the programs will be performed using mixed methods embedded in the case series. Tashakkori & Creswell describe mixed-methods research as “… research in which the investigator collects and analyses data, integrates the findings, and draws inferences using both qualitative and quantitative approaches or methods in a single study or program of inquiry” [16]. Quantitative data collection will be used for factual information, for instance the number of minutes spent per day on each treatment goal, as well as basic opinions such as the degree of confidence of parents in the cooperation with the therapist. Qualitative data collection will be used to gain understanding of in-depth experiences, for example regarding the experiences of parents with the program. In the process evaluation, the quantitative and qualitative data will be combined to draw conclusions regarding the processes of the home-based training programs.

## Methods

### COAD-study summary

The COAD-study consists of a comparative case series and a process evaluation. It is a multicentre study with a pragmatic nature, which will be performed in the Netherlands from April 2017 until October 2018. It is expected that participants will be enrolled from five rehabilitation centres on nine locations situated in both urban and rural areas. The study population consists of children aged 2 through 7 years with a clinically confirmed unilateral spastic CP and Manual Ability Classification System (MACS) level I-III [17], and their parents. A total of approximately 18 children and their parents will participate in the COAD-study. Children will be allocated to either the implicit home-based training program or the explicit home-based training program based on the preference of the parents. Parents receive an information leaflet regarding the difference between the programs and can discuss their decision with a health professional. We have described the protocols of the interventions in detail elsewhere [12].

The child-related primary outcome of the case series is performance of predetermined, individual rehabilitation goals, focused on bimanual daily life activities, as measured with the performance scale of the Canadian Occupational Performance Measure (COPM) [18]. With respect to the parents, therapy-related parental stress, explored with in-depth parental interviews, is of primary interest. Secondary outcomes focus on bimanual activity and participation of the child as well as (general) parental stress and empowerment.

### Design and paradigm

We will employ a mixed methods embedded design, that is a mixed methods approach in which quantitative as well as qualitative data collection and analyses are combined within a traditional quantitative or qualitative research design [19]. This study follows the embedded variant. The qualitative strand is implemented during the case series, thus a concurrent timing occurs. We consider the qualitative and quantitative parts of the case series and process evaluation equally important. The quantitative and qualitative strand will be interactive: mixing of methods occurred at the level of design and will continue during data collection (i.e. quantitative data will support selection of participants for elements of the qualitative strand) and during interpretation.

This study emerges from the pragmatism paradigm, which focuses on the consequences of actions, is problem centred, pluralistic and real-world practice oriented [19]. The qualitative strand involves a generic qualitative approach. Merriam describes generic qualitative research as an approach that cannot be specified as a particular type of qualitative study, such as grounded theory [20]. The aim of generic qualitative research studies is to understand the way people make sense of their lives and their experiences. However, it does not have an additional dimension that other designs have, such as understanding of a certain phenomenon in a phenomenological design [20].

### Home-based training programs

A multidisciplinary team of certified care providers, consisting of a paediatric physical or occupational therapist and a remedial educationalist or healthcare psychologist will deliver the programs. Each therapist will operate within only one home-based training program to prevent contamination. Allocation of a therapist to a program is based on the preference of the therapist. Remedial educationalists will operate across both programs. No contamination is expected, because remedial educationalists are instructed to avoid coaching with regard to the therapeutic content of the programs.

#### Course for therapists and remedial educationalists

Each therapist will complete a one-day course regarding the home-based training program. The course targets either the implicit approach or the explicit approach and mainly focuses on performing task analyses and designing an individualized home-based training plan in accordance with the specific learning approach. During a half-day course, the remedial educationalists will be informed on the content of both home-based training programs and instructed how to coach parents. Members of the research team who are experienced clinicians and educators will provide the courses. Since inclusion of participants within the centres will start consecutively, the courses are repeatedly delivered during the study. Refresher courses will be organized for therapists as well as remedial educationalists approximately one year after the initial training.

The home-based training programs consist of two phases: a preparation phase and the home-based training phase.

#### Preparation phase

The 2-week preparation phase involves four aspects. First, a blinded therapist who is not involved in the home-based training program of the child will determine five individual rehabilitation goals of importance to the parents and child, using the COPM [18]. Second, the coaching team gets acquainted with the parents and the child in an introductory meeting between the parents and the remedial educationalist and another meeting between the parents, child and coaching therapist, at the rehabilitation centre. During the latter meeting, the therapist will observe the manual abilities of the child and will videotape the child performing the activities that the rehabilitation goals comprise. Third, based on these videos the therapist will perform a task analysis. The task analysis approach is based on principles of stage 1 of the Perceive, Recall, Plan and Perform (PRPP) System of Task Analysis [21]. According to this task analysis, each task will be divided in steps. Thereafter, the error types in performance of each step, i.e. errors of omission, repetition, accuracy or timing, are registered. For participants in the explicit program, therapists additionally perform a movement analysis for each step in which errors occur. This movement analysis contains the posture and movement related actions the child does, as well as should perform in order to successfully complete the step. Subsequently, for all children therapists design an individualized training plan including instruction, feedback and organization of the task. Videos of the child during administration of the Assisting Hand Assessment (AHA) and the Observational Skills Assessment Score (OSAS) can facilitate training design [22, 23]. Fourth, the parents will be trained, which is twofold. Parents will receive instructional videos and a manual to study at home during the preparation phase. These materials address the content of the program, the coaching and teaching approach, and the use of the communication tool Quli (i.e. a Dutch online system for safe transfer of data such as documents, messages and videos between health care providers and health care consumers) [24]. Subsequently, at the end of the preparation phase, the therapist will visit the child and parents at home. This home visit allows the therapist to clarify the instructional videos, discuss the training plan, assess the physical home situation and available objects related to the rehabilitation goals, and answer parents’ questions. In case parents or the therapist have questions in the behavioural and social interaction domain, the remedial educationalist will be consulted. The manuals for therapists and remedial educationalists include various checklists, for example to guide the home visit.

#### Home-based training phase

The aim of the home-based training is improvement of the child’s performance of individual rehabilitation goals through training that is congruent with the context of the particular goal (i.e. task-specific therapy). This is in accordance with the latest version of the recommendations for care of children with spastic CP in the Netherlands, i.e. the ‘Richtlijn Spastische cerebrale parese bij kinderen’ [25]. During this phase, parents will provide training to their child in their home environment. The parent or caregiver most involved in the training will, as ‘primary trainer’, also have an active role in the data collection for the study. To foster implementation, a second parent or caregiver may also be involved in the training.

During the 12-week home-based training phase, parents and children will train for 3.5 h per week, preferably in meaningful situations. Parents can subdivide these training hours across the week in sessions with a minimum duration of 10 min.

In both programs, parents provide task-oriented and result-oriented instructions and feedback to the child. In the explicit approach, parents additionally give instructions and feedback to their child regarding specific motor execution of the tasks, whereas in the implicit approach parents provoke specific motor execution by the organization of the tasks.

Throughout the home-based training, parents will be coached by a therapist and a remedial educationalist. The parents and therapist will have a 30-min appointment over the phone weekly. In week 5 and week 9 the therapist will visit the parents at home for 60 min. If necessary, the therapist may schedule one additional home visit during the home-based training phase. Furthermore, parents will be contacted by phone by the remedial educationalist after the second week of home-based training. If requested by parents or therapist, basically one additional contact with the remedial educationalist can be planned.

To facilitate remote coaching, parents will register the amount and content of training they have performed with their child. Moreover, once a week they will record a training session on video. Parents will send the training registration form and the videos to the therapist and remedial educationalist via the communication tool Quli.

#### Follow-up period

A follow-up period succeeds the home-based training program, during which children will receive usual care. At the end of this 12-week period the final data will be collected to investigate the retention of training.

### Process evaluation methods

The approach described by Saunders et al. is used as the framework for the process evaluation. Saunders et al. describe five components of process evaluation: fidelity, dose, reach, recruitment, and context [26].

- *Fidelity* (quality) is defined as “the extent to which an intervention was implemented as planned”.
- *Dose* consists of dose delivered (completeness) and dose received (exposure and satisfaction). Dose delivered includes “the amount or number of intended units of each intervention or component delivered or provided by interventionists”. The exposure aspect of dose received is defined as “the extent to which participants actively engage with, interact with, are receptive to, and/or use materials or recommended resources”. The satisfaction aspect of dose received comprises “participant satisfaction with program, interactions with staff and/or investigators”.
- *Reach* (participation rate) is defined as “the proportion of the intended priority audience that participates in the intervention”.
- *Recruitment* comprises “procedures used to approach and attract participants at individual or organizational levels; includes maintenance of participant involvement in the intervention and measurement components of the study”.
- *Context* contains “aspects of the environment that may influence intervention implementation or study outcomes; includes contamination or the extent to which the control group was exposed to the program” [26].

Four of these five components of process evaluation will be investigated in our study. Reach of the home-based training programs in the context of the COAD-study is expected to be highly influenced by study related factors and will therefore not be assessed. Please note that throughout this paper implementation within the scope of the study is meant, not implementation within clinical practice.

#### Data collection

Table 1 shows the data collection methods, which will be used to assess the components fidelity, dose, recruitment and context of the process evaluation. The methods contain questionnaires, report and registration forms, videos, interviews and focus groups. In case of non-response to questionnaires and forms a maximum of three reminders will be send two, four and six days after the initial invitation. For the parent-related methods, the parent who is primary trainer will be asked to provide the requested input.

**Table 1:** Overview of data collection methods and respondents used to assess each process evaluation component per phase

			Process evaluation components					
Phase	Data collection method	Respondent	Fidelity	Dose delivered	Dose received (exposure)	Dose received (satisfaction)	Recruitment	Context
Course for therapists and remedial educationalists	Attendance				X			
	Questionnaire	Instructor	X	X				X
Preparation phase	Report form	Therapist	X					
Home-based training phase	Registration form	Parent			X			
	Videos	Parent	X					X
	Interviews	Parent	X			X		X
	Questionnaire	Therapists and remedial educationalists	X			X		X
Follow-up period	Interviews	Parent	X			X		X
Overall COAD home-based training programs	Focus groups	Therapists and remedial educationalists	X			X		X
	Drop-out: rates and reasons						X	

The following paragraphs will elaborate on the data collection.

##### Course for therapists and remedial educationalists

For each course, attendance of therapists and remedial educationalists will be registered.

The instructors of the therapists and remedial educationalists will evaluate the course by a digital questionnaire. The questionnaire includes questions regarding duration, location, content, positive and negative experiences, competence of the participants after the course, received feedback of the participants, and suggestions for improvement. All instructors are invited to fill out the questionnaire as soon as possible after the course and return it by e-mail.

##### Preparation phase

The first home visit will be evaluated by the therapist using a digital report form. This form includes for each point of the checklist for the first home visit provided by the protocol (as described in the paragraph ‘home-based training programs, preparation phase’) items regarding duration, understanding by parents, and particulars; other topics that were discussed; therapist’s impression of parents’ competence to execute the home-based training; and whether the remedial educationalist was going to be consulted instantly. The report form will be administered directly after the home visit by the online data collection application Castor EDC.

##### Home-based training phase and follow-up period

During the home-based training, all parents will register the daily amount of training and the kind of activities that were performed regarding each treatment goal (for example, closing the button of cotton trousers, while seated with the trousers on a table in front of the child); if applicable particular details per day, such as illness of the child; and perceived emotions of the parent as well as parent-rated emotions of the child related to the training via the use of emoticons. The digital registration form (Microsoft® Excel format) will be uploaded in the online data collection application Castor EDC by the parents at the end of the training program.

Besides, parents will be asked to make one video recording of a training session every week. From these twelve videos, six videos will be selected randomly. The video material will be scored by blinded assessors making use of a rating tool. The purpose of the tool is to define the degree of implicit and explicit approaches that parents use in training their child. This will be established by the proportion of task-oriented and result-oriented instruction/feedback versus instruction/feedback regarding motor execution of the task. A random selection of 20% of the videos will be rated in duplicate in order to calculate the inter-rater reliability of the tool.

Moreover, three in-depth parental interviews will be conducted: halfway the home-based training phase, after the home-based training has ended and after the follow-up period of 12 weeks. The interviews will be performed either with one parent or a parent-couple, including at least one parent actively engaged in the home-based training. The interviews will be semi-structured, following a pre-defined interview protocol. The duration of each interview will be approximately 60 min. To facilitate transferability of the different aims of the process evaluation, for the interviews a specific sub research question has been formulated: *How do parents experience the home-based training program and how do they integrate the program in the context of family life?* This question directs the interview process as well as data-analysis. Topics that will be covered during the first two interviews are: overall experience with the home-based training program; integrating the home-based training in the daily life of the child, the parent and the family; influence of the training on the parent and on the child; experiences of the parent as a co-therapist and perceived reactions of the child; the coaching by the health professional(s); and suggestions for improvement of the home-based training program. The third interview will cover retrospective experience with the home-based training program; (appreciation of) influence of the home-based training on the parent and the child after the intervention had ended; suggestions for improvement; retrospective considerations regarding participation in the home-based training program; and recommendation of the program to fellow parents. Additionally, therapy-related parental stress will be investigated by means of the interviews in the context of the case series of the COAD-study. The first interview will take place in person at the parents’ home, the second and third by video call. The interviews will be audiotaped. Trained interviewers will perform the interviews.

To evaluate the particular course of the home-based training program for each child, the therapist and remedial educationalist will each fill out a questionnaire. The questionnaires include questions regarding the execution and timing of program elements; content-related and procedural particularities; and their opinion on the application of the program by the parents. The questionnaires will be administered by the online data collection application Castor EDC, after the program of an individual child has ended.

##### Overall home-based training programs

Focus groups will be held with therapists and remedial educationalists involved in the study to explore their experiences with the home-based training programs. The health professionals will attend separate focus groups, based on their occupation and the home-based program they provided. Participation of all health professionals is desired, while practical difficulties to accomplish this are expected to result in a convenience sample. The duration of the focus groups will be 90–120 min. A topic list will guide the focus group discussions. This list will be designed based on the results of the other elements of the process evaluation. If a meeting in person is logistic not feasible, a simultaneous online focus group will take place. The focus groups will be videotaped. A trained researcher and assistant moderator will moderate the focus groups.

Dropout rates will be assessed for each home-based training program and, if available, reasons for drop-out will be recorded.

#### Data analysis

**Fig. 1:**
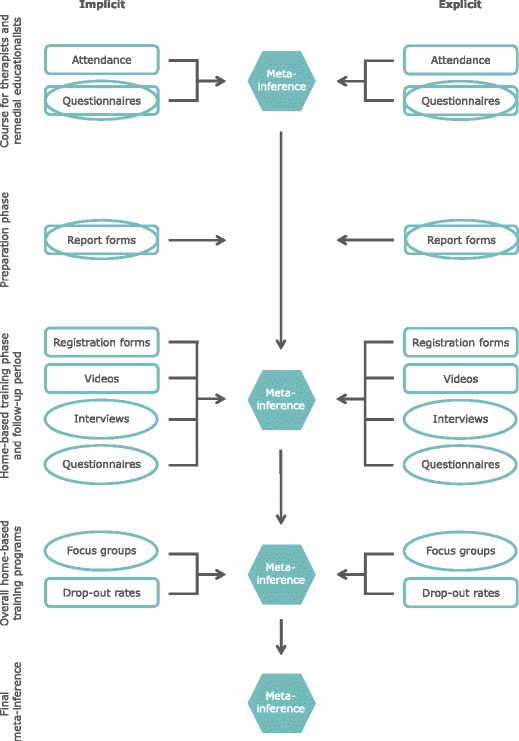
Overview data collection methods and the analysis process. Ovals represent primarily qualitative parts, rectangles primarily quantitative parts and hexagons meta-inferences

A graphical presentation of the data collection methods and the analysis process is given in Fig. 1.

Thematic analysis will be used for qualitative data, following the method described by Braun and Clarke [27, 28]. The analysis will be inductive (i.e. the identified themes will derive from the data) and on a latent/interpretative level. The latter indicates that ideas, assumptions, conceptualisations and ideologies will be determined that are theorized as underlying to the semantic content of the data. The first phase of the thematic analysis involves familiarization with the data. Verbatim transcripts will be created, the data will be read repeatedly and initial ideas will be noted. Phase 2 comprises systematic generation of initial codes. During phase 3 themes will be searched for by arranging codes into potential themes. Next, applicability of these themes to the coded extracts as well as the entire data set will be reviewed in phase 4. Phase 5 involves creating clear definitions and names for the themes, in order to refine their specifics. Phase 6 will offer the final opportunity for analysis. After selecting decent extract examples, final analysis and comparison of the analysis to the research aim and literature, the report will be produced [27, 28]. The qualitative data analysis software NVivo will be used throughout the data analysis.

Descriptive statistics include mean (standard deviation) or median (range) and number (%) for continuous and categorical data, respectively.

Initially the qualitative and quantitative data will be analysed concurrently. Thereafter, a side-by-side comparison will be performed using a summary table in which the qualitative and quantitative findings are merged. This meta-inference will be followed by interpretation of the combined results.

#### Validity and reliability of the qualitative strand

The process evaluation applies both between- and across-method triangulation by combining several quantitative and qualitative data-collection procedures, such as questionnaires, registration forms, in-depth interviews and focus groups. Data sources triangulation is performed by variance in respondents, namely trainers, parents, therapists and remedial educationalists. Two researchers will perform the data analysis. Hence, investigator triangulation occurs.

The results of the focus groups will be validated with the participating health professionals. No other member checking will be performed.

A researcher with expertise in qualitative research and with no other involvement in the project will peer review the process evaluation by verification of the analysis of 20% of the interviews and focus groups. In addition, she will critically analyse whether the conclusions are founded. By the peer review process, it is strived for sufficient independence in conducting the process evaluation and interpretation of its results.

#### Researcher bias and assumption

The interviews will be executed by LB, the data analysis will be performed by LB and MM. LB is a physical therapist by origin, currently she is a PhD candidate on the COAD-study. MM is a research assistant with a background as medical analyst. Because of the pragmatic nature of the study, the researchers involved in the process evaluation will remain passive observers during the study by avoiding interference with the home-based training programs and its delivery. Since the process evaluation will be executed by the project team, which is also responsible for development and delivery of the programs and for the evaluation of outcomes of the case series, peer review will be performed as described in the previous paragraph.

The study, including data analysis, will be performed in Dutch. The findings and supporting evidence will be translated into English. Back translation of 20% of this material will be executed to increase credibility.

## Discussion

This protocol outlines the background and design of the process evaluation of two home-based bimanual training programs for children with uCP. Evaluating two programs that differ regarding the approach by which parents teach motor activities to their child is innovative. Comparing an implicit with an explicit approach originates from the demand to increase bimanual activity and participation of the child whilst minimizing parental stress as a result of home-based training. Furthermore, to our knowledge, this is the first study to perform an extensive process evaluation parallel to an effect evaluation regarding home-based training programs in paediatric rehabilitation. The aim of the process evaluation is to assess fidelity of the home-based training programs, to examine the mechanisms that cause the relation between the programs and their effects, and to determine the influence of contextual factors.

A key strength of the process evaluation is the use of mixed methods. In general, it is assumed that triangulation of quantitative and qualitative methods leads to greater validity than either single one alone, and that combining them offsets the weaknesses of each individual method [19]. For this specific study, the mixed methods design is chosen to get a more complete understanding of processes occurring with regard to the home-based training programs and experiences of parents with the programs. Moreover, the qualitative data are expected to facilitate the explanation of the quantitative findings. The use of different types of triangulation as well as data collection during all phases of the home-based training programs will enhance the credibility (i.e. internal validity) of the study.

Member checking is limited to the focus groups at the end of the study. Member checking throughout the study would be expected to influence behaviour of parents, therapists and remedial educationalists during the home-based training programs, possibly affecting the results of the process and/or effect evaluation. Another limitation includes execution of the process evaluation by the project team itself, which may introduce researcher bias. However, acknowledging this possibility as well as peer review by an independent researcher is assumed to reduce this risk.

The findings of this process evaluation will have implications for clinical practice and further research regarding development and application of home-based bimanual training programs, executed by parents and aimed at improving activity performance and participation of children with uCP.

## Abbreviations

AHA: Assisting Hand Assessment
Castor EDC: Castor Electronic Data Capture
COAD: Co-creation a hand: the road to independence
COPM: Canadian Occupational Performance Measure
CP: Cerebral Palsy
MACS: Manual Ability Classification System
OSAS: Observational Skills Assessment Score
PRPP: Perceive, Recall, Plan and Perform
uCP: Unilateral Cerebral Palsy
